# Large cap-polyposis of the sigmoid successfully treated with endoscopic electroporation

**DOI:** 10.1055/a-2643-8800

**Published:** 2025-08-01

**Authors:** Romano Sassatelli, Cristina Manzotti, Giuliana Sereni, Veronica Iori, Maurizio Cavina, Loredana De Marco, Fabio Bassi

**Affiliations:** 1 9242 Gastroenterology and Digestive Endoscopy Unit, AUSL-IRCCS di Reggio Emilia, Reggio Emilia, Italy; 2 9242 Pathology Unit, AUSL-IRCCS di Reggio Emilia, Reggio Emilia, Italy

A 77-year-old female underwent a colonoscopy for anemia and hematochezia. A 40-mm polyp of the sigmoid colon with digitiform estroflexions and bleeding erosions covered by fibrin sheats was found, within an hyperemic, edematous mucosa with multiple diverticula ( Fig. 1 ). Biopsies revealed hyperplastic mucosa with tortuous, elongated crypts and abundant inflammation in the lamina propria, with characteristic “cap” and mucus on the surface, suggesting a diagnosis of CAP polyposis ( Fig. 2 ). Owing to a large symptomatic although benign lesion, a treatment with electroporation was decided.

**Fig. 1:**
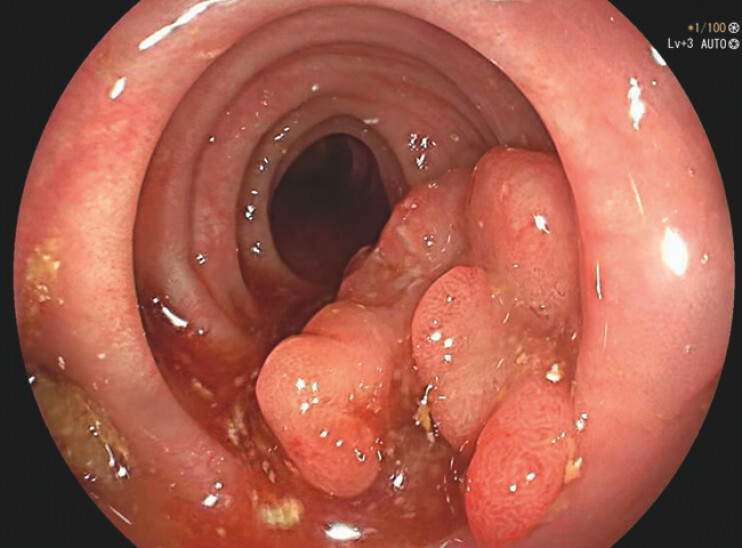
40-mm CAP polyposis of the sigmoid at diagnosis – endoscopic image.

**Fig. 2:**
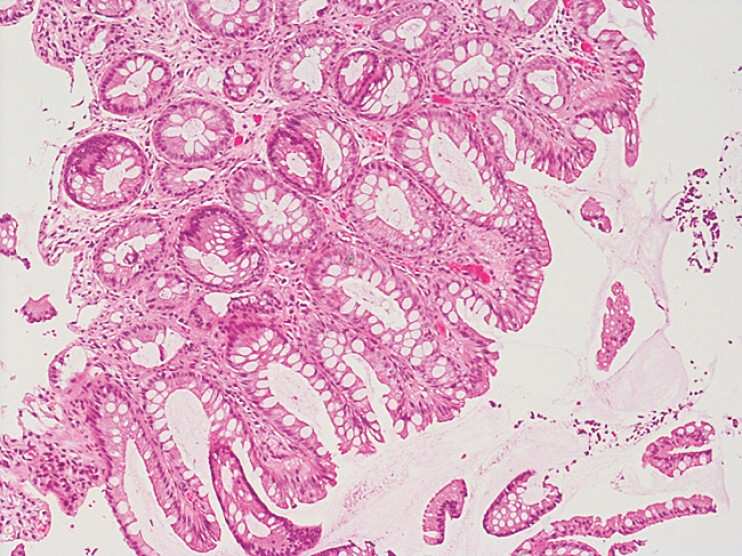
Hystological examination. Hyperplastic mucosa with tortuous, elongated crypts and abundant inflammation in the lamina propria, with characteristic “cap” and mucus on the mucosal surface.

After submucosal injection of calcium gluconate, endoscopic electroporation with EndoVE was performed ( Video 1 ), with 25 applications, without adverse events. A 1-month colonoscopy showed a scar area ( Fig. 3 ) with no relapse at biopsies, and a 6-month colonoscopy confirmed no relapse ( Fig. 4 ).

Endoscopic electroporation with EndoVE of CAP polyposis of the sigmoid colon, after submucosal injection with calcium gluconate.Video 1

**Fig. 3:**
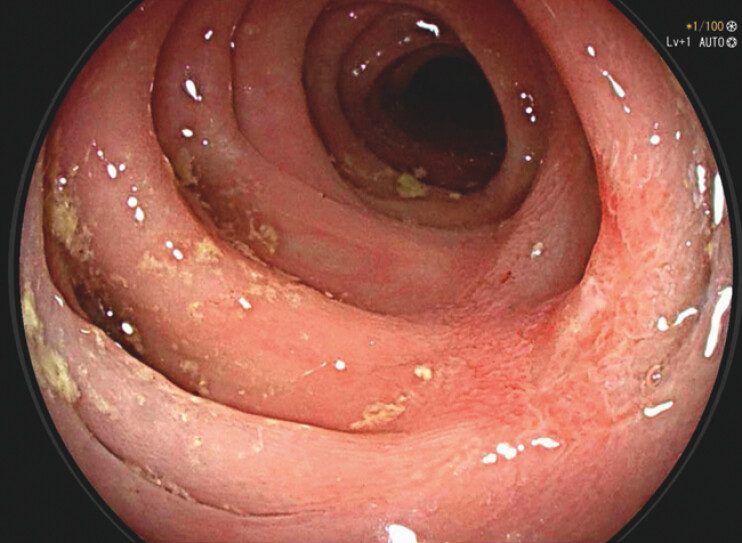
1-month colonoscop – scar with no relapse.

**Fig. 4:**
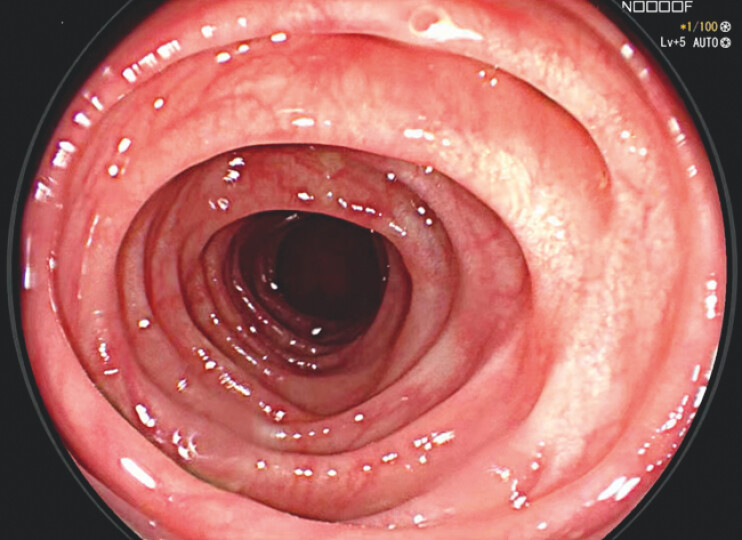
6-months colonoscopy – no relapse.

To our knowledge, this is the first description of a CAP polyposis treated with endoscopic electroporation, with successful results.

Electroporation uses electric pulses that enhance cell permeability and conductivity, facilitating the transport of molecules or inducing cell necrosis, depending on cell characteristics and electrical parameters applied. Its combination with different substances injection (such as calcium gluconate or other chemical agents)has been tested as a promising ablation technique for anticancer treatment in preclinical and clinical studies 1 2 3 4 .

EndoVE is designed to perform endoscopic electroporation on gastrointestinal tissue. It can be used to treat solid tumours within the GI tract, utilizing a vacuum to draw the lesion into contact with electrodes, delivering a pulsed electrical field.

Cap polyposis is a rare disease with an unclear pathogenesis, characterized by erythematous, inflammatory polyps of the rectum or sigma, often with an adherent fibrin sheath (“cap”). Patients usually present with mucoid diarrhea or rectal bleeding. Multiple treatments for CAP polyposis have been proposed, such as pharmacological treatments (aminosalicylates, steroids, metronidazole, H. pylori eradication therapy, infliximab), endoscopic resection, and surgery for refractory cases, but with variable results and without standardized management defined 5 .

In our case of a large bleeding CAP polyposis, electroporation with EndoVE was a safe, conservative, and successful treatment. Further studies are needed to confirm its safety and efficacy for the treatment of GI lesions.

Endoscopy_UCTN_Code_TTT_1AQ_2AD

## Notes

Endoscopy E-Videos https://eref.thieme.de/e-videos
*E-Videos* is an open access online section of the journal *Endoscopy* , reporting on interesting cases and new techniques in gastroenterological endoscopy. All papers include a high-quality video and are published with a Creative Commons CC-BY license. Endoscopy E-Videos qualify for HINARI discounts and waivers and eligibility is automatically checked during the submission process. We grant 100% waivers to articles whose corresponding authors are based in Group A countries and 50% waivers to those who are based in Group B countries as classified by Research4Life (see: https://www.research4life.org/access/eligibility/ ). This section has its own submission website at https://mc.manuscriptcentral.com/e-videos .
